# Molecular characterization and multi-locus genotypes of *Enterocytozoon bieneusi* from captive red kangaroos (*Macropus Rfus*) in Jiangsu province, China

**DOI:** 10.1371/journal.pone.0183249

**Published:** 2017-08-14

**Authors:** Zhijun Zhong, Yinan Tian, Yuan Song, Lei Deng, Junxian Li, Zhihua Ren, Xiaoping Ma, Xiaobin Gu, Changliang He, Yi Geng, Guangneng Peng

**Affiliations:** 1 Key Laboratory of Animal Disease and Human Health of Sichuan Province, College of Veterinary Medicine, Sichuan Agricultural University, Chengdu, Sichuan, P.R. China; 2 Nanjing Hongshan Forest Zoo, Nanjing, Jiangsu, P.R. China; University of Minnesota, UNITED STATES

## Abstract

*Enterocytozoon bieneusi* is the most common pathogen of microsporidian species infecting humans worldwide. Although *E*. *bieneusi* has been found in a variety of animal hosts, information on the presence of *E*. *bieneusi* in captive kangaroos in China is limited. The present study was aimed at determining the occurrence and genetic diversity of *E*. *bieneusi* in captive kangaroos. A total of 61 fecal specimens (38 from red kangaroos and 23 from grey kangaroos) were collected from Nanjing Hongshan Forest Zoo and Hongshan Kangaroo Breeding Research Base, Jiangsu province, China. Using the nested PCR amplification ITS gene of rRNA of *E*. *bieneusi*, totally 23.0% (14/61) of tested samples were PCR-positive with three genotypes (i.e. one known genotype, CHK1, and two novel genotypes, CSK1 and CSK2). Multi-locus sequence typing using three microsatellites (MS1, MS3, and MS7) and one minisatellite (MS4) revealed one, five, two, and one types at these four loci, respectively. In phylogenetic analysis, the two genotypes, CHK1 and CSK1, were clustered into a new group of unknown zoonotic potential, and the novel genotype CSK2 was clustered into a separate clade with PtEb and PtEbIX. To date, this is the first report on the presence of *E*. *bieneusi* in captive red kangaroos in Jiangsu province, China. Furthermore, a high degree of genetic diversity was observed in the *E*. *bieneusi* genotype and seven MLGs (MLG1-7) were found in red kangaroos. Our findings suggest that infected kangaroo may act as potential reservoirs of *E*. *bieneusi* and be source to transmit infections to other animal.

## Introduction

*Enterocytozoon bieneusi* is an unicellular enteric microsporidian that causes diarrhea in a variety of domestic and wild animals worldwide as well as in humans, especially in children and immunosuppressed individuals [1]. Since *E*. *bieneusi* was first found in enterocytes of Haitian patients with AIDS and then identified in pig feces, many studies were concentrated on the role of other animals as reservoirs in the epidemiology of this pathogen [2, 3]. Animals infected with *E*. *bieneusi* can shed spores through feces into the environment, which can contaminate food or water, leading to microsporidiosis outbreaks [4]. To date, more than 240 genotypes of *E*. *bieneusi* have been identified based on the analysis of the sequences of the single internal transcribed spacer (ITS) region of the rRNA gene [5–8]. These genotypes of *E*. *bieneusi* have been divided into nine different groups based on phylogenetic analysis [9, 10]. Group 1, which is usually considered as a human-pathogen group, contains 94% of all the identified ITS genotypes of *E*. *bieneusi*, and the remaining eight major clusters (groups 2 to 9) are found mostly in specific hosts and wastewater [7, 11].

The genetic identity of *E*. *bieneusi* has been widely investigated in several domestic and wild animals in China, but reports on *E*. *bieneusi* in captive kangaroos are limited [12, 13]. Only one study so far has reported *E*. *bieneusi* infection in white kangaroos and grey kangaroos in the Zhengzhou zoo in China [12], and there are no reports on *E*. *bieneusi* infection in red kangaroos in China. The Nanjing Hongshan Forest Zoo and the Hongshan Kangaroo Breeding Research Base are the dominating kangaroo breeding bases in China, from where the kangaroos are mainly taken to other zoos as ornamental animals. Because the kangaroos are mainly maintained in zoos and their feces directly defecate to the environment, infectious spores of *E*. *bieneusi* from these kangaroos can be transmitted to other animals and even pose a threat to public health. Therefore, this study was aimed at examining the infection rate and genetic diversity of *E*. *bieneusi* in captive kangaroos from Jiangsu province in China using ITS sequencing and multilocus sequence typing (MLST) analysis.

## Methods

### Ethic statement

This study was reviewed and approved by the Research Ethics Committee and the Animal Ethical Committee of Sichuan Agricultural University. Appropriate permission was obtained from zoo managers before the collection of fecal specimens. During the collection of fecal specimens, the animals were not subjected to any kind of injury.

### Specimen collection

A total of 61 fecal specimens (38 from red kangaroos and 23 from grey kangaroos) were collected between November 2016 and January 2017 from captive kangaroos at the Nanjing Hongshan Forest Zoo and Hongshan Kangaroo Breeding Research Base in Jiangsu province, China (Table 1). Fresh fecal specimens (approximately 10 g) from each kangaroo were collected immediately by feeders after defecation on the ground and then quickly transferred into individual 50-mL plastic containers. All the kangaroos showed no obvious clinical symptoms at the time of sampling. All the fecal specimens were stored at 4°C in 2.5% (w/v) potassium dichromate until use.

**Table 1:** Prevalence and distribution of *E*. *bieneusi* genotypes in Nanjing Hongshan Forest Zoo and Hongshan Kangaroo Breeding Research Base.

Characteristics	No. examined	No. positive (%)	Genotype
Zoo			
Nanjing Hongshan Forest Zoo	34	0 (0%)	
Hongshan Kangaroo Breeding Research Base	27	14 (51.9%)	CHK1, CSK1, CSK2
Species			
Red kangaroo	38	14 (36.8%)	CHK1, CSK1, CSK2
Grey kangaroo	23	0 (0%)	
Gender			
Male	19	3 (15.7%)	CHK1
Female	42	11 (26.2%)	CHK1, CSK1, CSK2

### DNA extraction

Genomic DNA was extracted from 200 mg of each fecal sample using the EZNA® Stool DNA kit (Omega Biotek, Norcross, GA, USA) according to the manufacturer’s protocol. The DNA was then eluted in 200 μL of absolute ethanol and stored at -20°C prior to PCR analysis.

### PCR amplification

All the DNA preparations were analyzed for the presence of *E*. *bieneusi* using nested PCR amplification of a 389-bp nucleotide fragment of the rRNA gene. Positive specimens were further characterized by MLST analyses using the MS1, MS3, MS4, and MS7 loci. The primers and cycling parameters employed for these reactions were as previously described [14, 15]. TaKaRa Taq DNA Polymerase (TaKaRa Bio Inc., Tokyo, Japan) was used for all the PCR amplifications. A negative control with no DNA added was included in all the PCR tests. All the secondary PCR products were subjected to electrophoresis on a 1.5% agarose gel and visualized by staining the gel with Goldenview.

### Nucleotide sequencing and analysis

All the secondary PCR products of the expected size were directly sequenced with a set of primers used for the secondary PCR by Life Technologies (Guangzhou, China) using a BigDye® Terminator v3.1 cycle sequencing kit (Applied Biosystems, Carlsbad, CA, USA).

The nucleotide sequences obtained in this study were aligned with each other and with reference sequences downloaded from GenBank using the Basic Local Alignment Search Tool (BLAST) (http://www.ncbi.nlm.nih.gov/BLAST/) and ClustalX 1.83 (http://www.clustal.org/) to determine the *E*. *bieneusi* genotypes. The genotypes were assigned previously published names if found to be identical to known genotypes. Genotypes with single nucleotide substitutions, deletions, or insertions in 243 bp of the ITS gene region of *E*. *bieneusi* relative to the those of the known genotypes were considered novel genotypes and named according to the established nomenclature system [16].

### Phylogenetic analysis

To better assess the diversity of the *E*. *bieneusi* genotypes in the present study and to determine the genetic relationship between the novel ones isolated from the kangaroos and the reference sequences previously published in GenBank, phylogenetic analysis was performed by constructing a neighboring-joining tree using Mega 6 software (http://www.megasoftware.net/), which is based on evolutionary distances calculated using a Kimura 2-parameter model. The reliability of these trees was assessed using bootstrap analysis with 1,000 replicates. The topology tree was constructed by a maximum parsimony analysis of the same sequence alignment, with all alignment sites taken into consideration[17].

### Nucleotide sequence accession numbers

Representative nucleotide sequences were deposited into the GenBank database under the following accession numbers: KY706126 to KY706128 for the rRNA gene ITS sequences of *E*. *bieneusi*, and KY706117 to KY706125 for the microsatellite loci (MS1, MS3, and MS7) and minisatellite (MS4).

## Results and discussion

In this study, 14 (23.0%) out of 61 captive kangaroos were found to be infected with *E*. *bieneusi*, and all the positive specimens belonged to red kangaroos from the Hongshan Kangaroo Breeding Research Base, which acts as the largest kangaroo breeding zoo in China. The male and female kangaroos showed an infection rate of 15.7% and 26.2%, respectively, with no significant difference (P *>* 0.05) (Table 1). The overall infection rate was lower than that observed in captive Pere David’s deer (34.0%, 16/47) in the Henan province of China and in captive black bear (27.4%, 29/106) in the Sichuan province of China [18, 19], but higher than the rates reported for captive wildlife in the Zhengzhou Zoo (15.8%) and for pet chinchillas (3.6%) in China [12, 20].

**Fig 1:**
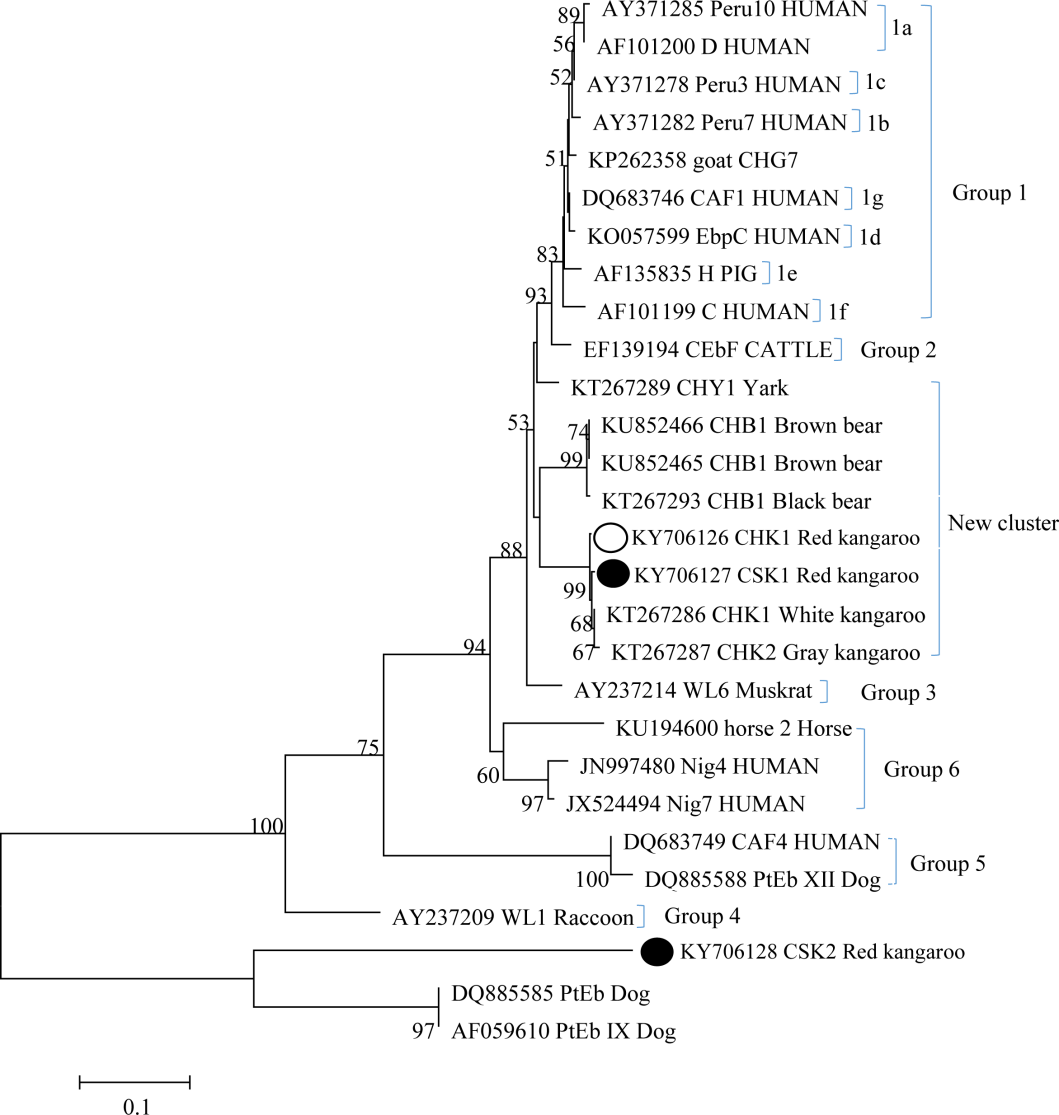
Phylogenetic relationship among the ITS loci of *E*.*bieneusi* isolates. Phylogenetic relationship of the genotypes of *E*. *bieneusi* identified in this study and known genotypes previously published in GenBank as inferred by a neighbor-joining analysis of ITS sequences based on genetic distances calculated by the Kimura 2-parameter model. A similar topology tree was also performed by maximum parsimony analysis, with the exception that the CSK2 genotype grouped together with genotypes PtEb and PtEbIX, with 99% bootstrap value. The numbers on the branches are percent bootstrapping values from 1000 replicates, with more than 50% shown in the tree. Each sequence is identified by its accession number, genotype designation, and host origin. Genotypes marked with black circles and open circle are novel and known genotypes identified in this study, respectively.

Sequence analysis of the amplified PCR products revealed three ITS genotypes: a known genotype, CHK1, and two novel genotypes, CSK1 and CSK2. The known genotype, CHK1, has been identified previously in white kangaroos at the Zhengzhou zoo [12], and the novel genotype, CSK1, has 20 bases different from AF267144; the other novel genotype, CSK2, has higher genetic variability than the genotype KIN-3 (JQ437575). Phylogenetic analysis also showed that the genotypes CHK1 and CSK1 were clustered into a new group, which was first reported by our laboratory [6], and this group contains genotypes almost from bears and kangaroos [12]. The novel CSK2 genotype was grouped in a separate cluster together with genotypes PtEb IX and PtEb from dogs[21] (Fig 1). Genotypes of the new group have been detected in different animals in the past; however, whether these genotypes have the potential for transmission across species needs to be investigated in the future. Furthermore, the origin of *E*. *bieneusi* in the kangaroo population is not clear. All kangaroos in Hongshan Kangaroo Breeding Research Base are imported from Australia through Kangaroos Exchanging Program between China and Australia. However, no report for *E*. *bieneusi* infection in kangaroos in Australia, indicating the *E*. *bieneusi* infection in kangaroos is likely introduced in China. Thus further work should focus on the occurrence of these protest in other kangaroos population as well as other animals in China.

MLST using MS1, MS3, MS4, and MS7 has been developed for studying the taxonomy and population genetics of *E*. *bieneusi* [22]. Recently, high multilocus genotype (MLG) diversity has been observed for genotypes such as I-like, I, J, CHB1, SC01, BEB6, D, and horse1 that have the same ITS gene sequences [6, 10, 23–25]. In this study, all the ITS-positive samples were amplified and sequenced at the four loci. A total of 11, 13, 11, and 12 fecal samples were successfully amplified at the MS1, MS3, MS4, and MS7 loci, respectively, but only 10 samples were simultaneously positive at all four loci (S1 Table). Sequence analysis revealed one, five, two, and one genotypes at the MS1, MS3, MS4, and MS7 loci, respectively, and seven distinct MLGs (MLG1–7) were observed in genotype CHK1. Thus, our findings revealed the high genetic diversity of genotype CHK1 of *E*. *bieneusi* in kangaroos.

In conclusion, this is the first report on *E*. *bieneusi* infection in captive red kangaroos in Jiangsu province, China. Two novels genotypes (CSK1 and CSK2) were identified by analysis of the ITS gene. Genetic diversity was observed in genotype CHK1 using the MLST tool, and seven MLGs were found in red kangaroos. Because of the high-density feeding environment in zoos and the lack of systematic deal feces method from animals, proper advice should be given to the managers of the Kangaroo Breeding Research Base to take steps to avoid interspecies transmission of *E*. *bieneusi*.

## Supporting information

S1 Table Multi-locus sequence typing of *Enterocytozoon bieneusi* in red kangaroos in Hongshan Kangaroo Breeding Research Base, Jiangsu province, China. (DOCX)
