# Correction: Molecular characterization and multi-locus genotypes of *Enterocytozoon bieneusi* from captive red kangaroos (*Macropus Rfus*) in Jiangsu province, China

**DOI:** 10.1371/journal.pone.0190660

**Published:** 2017-12-29

**Authors:** Zhijun Zhong, Yinan Tian, Yuan Song, Lei Deng, Junxian Li, Zhihua Ren, Xiaoping Ma, Xiaobin Gu, Changliang He, Yi Geng, Guangneng Peng

The word “*Rufus*” is misspelled in the article title. The correct title is: Molecular characterization and multi-locus genotypes of *Enterocytozoon bieneusi* from captive red kangaroos (*Macropus Rufus*) in Jiangsu province, China. The correct citation is: Zhong Z, Tian Y, Song Y, Deng L, Li J, Ren Z, et al. (2017) Molecular characterization and multi-locus genotypes of *Enterocytozoon bieneusi* from captive red kangaroos (*Macropus Rufus*) in Jiangsu province, China. PLoS ONE 12(8): e0183249. https://doi.org/10.1371/journal.pone.0183249
