# Supplementary material for: Molecular characterization and multi-locus genotypes of Enterocytozoon bieneusi from captive red kangaroos (Macropus Rfus) in Jiangsu province, China
Source: PLoS One. 2017 Aug 14;12(8):e0183249. doi: 10.1371/journal.pone.0183249 (PMC5555684; doi:10.1371/journal.pone.0183249)
Supplement: S1 Table — (DOCX) [file pone.0183249.s001.docx]

S1 Table. **Multi-locus sequence typing of *Enterocytozoon bieneusi* in red kangaroos in Hongshan Kangaroo Breeding Research Base, Jiangsu province, China**

| ITS genotype | MS1 | MS3 | MS4 | MS7 | MLGs |
| --- | --- | --- | --- | --- | --- |
| CHK1 | Type I | Type I | Type I | Type I | MLG1 |
| CHK1 | Type I | Type I | Type I | Type I | MLG1 |
| CHK1 | Type I | Type I | Type II | Type I | MLG2 |
| CHK1 | Type I | Type II | Type I | Type I | MLG3 |
| CHK1 | Type I | Type II | Type I | Type I | MLG3 |
| CHK1 | Type I | Type IV | Type I | Type I | MLG4 |
| CHK1 | NA | Type III | NA | NA | NA |
| CHK1 | Type I | Type III | Type I | Type I | MLG5 |
| CHK1 | Type I | Type V | Type I | Type I | MLG6 |
| CHK1 | Type I | Type I | Type I | Type I | MLG1 |
| CSK1 | NA | Type II | NA | Type I | NA |
| CHK1 | Type I | Type II | NA | Type I | NA |
| CSK2 | NA | NA | Type I | NA | NA |
| CHK1 | Type I | Type II | Type II | Type I | MLG7 |

Note: NA = not amplified
